# Associations of statin use with motor performance and myalgia may be modified by 25-hydroxyvitamin D: findings from a British birth cohort

**DOI:** 10.1038/s41598-017-06019-z

**Published:** 2017-07-26

**Authors:** Nikhil Sharma, Rachel Cooper, Diana Kuh, Imran Shah

**Affiliations:** MRC Unit for Lifelong Health and Ageing at UCL, 33 Bedford Place, London, WC1B 5JU UK

## Abstract

The objective was to examine whether: (1) statin use was associated with muscle related outcomes at age 60–64, (2) these associations were modified by 25-hydroxyvitamin D (25(OH)D) status and explained by inflammation, body-size or lifestyle in a British birth cohort. Markers of myalgia (intrusive body pain) and myopathy (self-reported and performance-based measures) were examined in 734 men and 822 women (MRC National Survey of Health and Development). Statin use was associated with intrusive body pain, difficulty climbing stairs and slower chair rise speed. Some associations were modified by 25(OH)D e.g. the association with intrusive body pain was evident in the insufficient (13–20 ng/l) and deficient (<13 ng/l) 25(OH)D status groups (OR = 2.6,95% CI 1.7–1.1; OR = 1.8,95% CI 1.2–2.8, respectively) but not in those with status >20 ng/l (OR = 0.8,95% CI 0.5–1.4) (p = 0.003 for interaction). Associations were maintained in fully adjusted models of intrusive body pain and difficulty climbing stairs, but for chair rise speed they were fully accounted for by inflammation, body-size and lifestyle. In a nationally representative British population in early old age, statin use was associated with lower limb muscle-related outcomes, and some were only apparent in those with 25(OH)D status below 20 ng/l. Given 25(OH)D is modifiable in clinical practice, future studies should consider the links between 25(OH)D status and muscle related outcomes.

## Introduction

There is clear and compelling evidence that statins have a beneficial effect on cardiovascular risk factors^1,2^. This is reflected in recent guidelines from NICE^3^ that suggest that statins should be prescribed to anyone with a 10% ten-year risk of a cardiovascular event. As a result, approximately one-quarter of the population aged between 30 and 85 years old could be prescribed a statin. However, there remain serious concerns about the potential adverse effects of statins if prescribed to such a large population^4,5^.

There is a discrepancy between the data from randomised controlled trials (RCT) and observational studies, therefore the incidence of adverse effects of statins continues to be debated^6^. Data from large randomised controlled trials (RCT) suggest that adverse effects are similar to placebo. In a review of the trial data, Finegold (2014) concluded that only a minority of symptoms were genuinely due to statin use^7^. Nevertheless, it should be noted that the trials do not typically monitor for more indolent muscle conditions. For instance, the influential CTT collaboration meta-analysis of trials explored a small set of adverse effects that only included acute muscle conditions (vascular events, cancers, rhabdomyolysis and deaths)^1^. As chronic muscle conditions have not generally been recorded in RCTs, it cannot be assumed that an absence of evidence is evidence of absence.

Observational studies of patients on statins typically focus on chronic muscle conditions and report a much higher rate of adverse effects than the RCTs. In a retrospective study of patients in routine care in Boston (USA), myalgia (muscle pain) and myopathy (muscle weakness) were the most commonly documented reasons for statin discontinuation, affecting 27% of patients^8^. Similarly, in a survey of over ten thousand statin users, a quarter of users reported muscle related side effects^9^. Cross sectional population data from the National Health and Nutrition Examination Survey (NHANES 1999–2002) suggest 22% of participants taking a statin reported musco-skeletal pain compared with 18% participants not taking a statin^10^. Other large observational studies, some with up to 9 years of follow up^11^, report a higher prevalence of muscle related events in statin users compared with non statin users^12^. Data from UK general practices are unclear; there are reports of an increased risk of moderate to severe myopathic events with statin use^13^ but other reports show no difference in myositis between statin users and non users^14^. The few studies to include measures of motor performance did not find an association between statin use and reduced physical performance^15–18^ or response to physical training^19^. In both the clinical trials and observational studies there is little consistency in what constitutes a statin related adverse effect which makes direct comparison challenging.

There are many additional factors that could contribute to an increased risk of muscle related conditions in statin users compared with non users in observational studies. For instance, low 25-hydroxyvitamin D is prevalent in the UK population^20,21^ and is a cause of myopathy and myalgia. Previous studies report that low 25-hydroxyvitamin D status may exacerbate musculoskeletal symptoms experienced with statin use^22–24^. Indeed, in a small series of 128 patients with statin induced myalgia, 25-hydroxyvitamin D was significantly lower in statin users than non users and responded positively to replacement in the majority of cases^25^. Overall this highlights the potential interaction between low 25-hydroxyvitamin D status and statin use in relation to adverse effects.

Higher body mass index, adverse lifestyle factors such as smoking, and socioeconomic conditions (SEC) are associated with muscle-related outcomes and have been shown to be associated with 25-hydroxyvitamin D levels^26,27^. Statin users tend to have higher levels of inflammatory markers such as Interleukin 6 (IL-6) and C-reactive protein (CRP) and elevated levels of these markers have also been associated with poorer motor performance^28^, strength^29^, BMI^30^ and lifestyle^31^.

We investigated whether statin use was associated with a range of muscle related outcomes in a large nationally representative British birth cohort of men and women followed up to early old age. We assessed intrusive body pain as a marker of myalgia. In addition, we used both self-reported and performance-based measures of limb function as markers of myopathy. We hypothesized that any association between statin use and these outcomes would be modified by current 25-hydroxyvitamin D (based upon published data refs^22–24^) and accounted for by inflammatory status, prior body mass index (BMI) and osteoarthritis (OA), smoking and SEC^32^. Testing these hypotheses in a nationally representative British cohort addresses a significant gap within the literature that directly informs the implementation of the NICE guidelines.

## Methods

### Study population

The MRC National Survey of Health and Development (NSHD) has followed a nationally representative sample, originally of 5,362 individuals, born in mainland Britain in March 1946. At the 23rd follow up at 60–64 years, study members first received postal questionnaires to complete and were then invited to attend one of six clinical research facilities (CRFs) or to have a research nurse visit their home^33^. Of the 3163 people in the target sample, information was obtained from 2662 (84%) participants, of whom 2474 completed a postal questionnaire and 2229 had a clinical assessment (1690 at a CRF and 539 at home). No attempt was made to contact the remaining study members: 718 (13.4%) had already died, 594 (11.1%) had previously withdrawn from the study, 567 (10.6%) lived abroad and 320 (5.9%) had been untraceable for more than ten years. The study received ethical approval for the data collection at age 60–64 from the Central Manchester Local Research Ethics Committee and the Scotland A Research Ethics Committee and is compliant with the appropriate guidelines and regulations. Informed consent was provided by participants.

### Intrusive Body pain

The definition of myalgia is broad. We used a question from the SF-36 (included on the postal questionnaire) that asked about intrusive body pain (based on how much the pain interfered with normal work including both work outside the home and housework)^34^. The data for responses were recoded from a scale of 0 (not at all) to 4 (extremely) into a binary variable which indicated if intrusive body pain was absent (0) or present (1–4).

### Limb function

Muscle disorders predominantly affect the proximal muscle groups. We therefore used measures that represent proximal limb function and compared them with measures of distal limb function. Assessments of function were conducted during the clinical assessment at age 60–64 with trained nurses following standardised protocols for the performance-based tests^35^.

### Difficulties going up/down stairs

As a subjective indicator of proximal muscle function participants were asked if they had difficulty going up and down a flight of 12 stairs (coded Yes vs No)^36^.

### Difficulties gripping heavy objects

Participants were asked if they had difficulty gripping heavy objects like a full kettle (coded Yes vs No). This was used as a subjective indicator of distal limb function.

### Chair Rise speed

Chair rise speed was used as an objective indicator of proximal lower limb function. Chair rise speed per minute was calculated from the time taken to rise from a sitting to a standing position with straight back and legs, and then sit down again as fast as possible ten times (chair rise speed per minute = (10/chair rise time)*60). Where a test was not completed for reasons of poor health (n = 136), values were imputed as in previous analyses^37,38^, by taking the sex-specific mean of the bottom fifth.

### Grip strength

Grip strength was used as an objective indicator of distal limb function and was measured isometrically using an electronic handgrip dynamometer^39^. The maximum strength achieved during 6 attempts (three in each hand) was used. Where a test was not completed for reasons of poor health (n = 49), values were imputed as in previous analyses^37,38^, by taking the sex-specific mean of the bottom fifth.

### Statin use

Study members were asked to report all regularly prescribed medications on the postal questionnaire and by the nurse at their clinical assessment. These reports were coded according to the British National Formulary (BNF) formulary and a variable denoting statin use (yes vs no) was derived.

### Current 25-hydroxyvitamin D and inflammatory status

Overnight fasting blood samples were taken during the clinical assessment and initially processed at the CRF laboratories. Aliquots were frozen and stored prior to being transferred to the MRC Human Nutrition Research (HNR) laboratory in Cambridge where analyses of CRP was processed according to standardised protocols^40^. Analyses of IL-6 and 25-hydroxyvitamin D were undertaken by the British Heart Foundation Research Centre in Glasgow (BHFRC) the latter using LC MS/MS (Liquid chromatography–mass spectrometry). Inflammatory markers were positively skewed and therefore transformed using the natural logarithm to achieve normality of their distributions^40^ and used as continuous variables.

We carefully considered how to model 25-hydroxyvitamin D in these analyses and despite the acknowledged loss of statistical power we decided *a priori* to adopt a categorical approach for the following reasons. First, supporting literature, particularly the guidelines for clinicians (for instance the US reference ranges^41^), typically consider 25-hydroxyvitamin D as categories. Second, treating 25-hydroxyvitamin D as a continuous variable assumes interactions are linear and ignores the cellular^42^, observational^43^ and clinical^44^ evidence that supports a threshold effect of 25-hydroxyvitamin D status.

Therefore 25-hydroxyvitamin D levels were split into thirds; normal (>20 ng/l); insufficient (13–20 ng/l); and deficient (<13 ng/l). This is broadly similar to the US reference ranges (insufficient 25-hydroxyvitamin D is considered <12 ng/l). A priori, based upon published data^22–24^, we hypothesized that the effect of statin on the muscle outcomes would be exacerbated by low 25-hydroxyvitamin D.

### Other covariates

We included those covariates that have been shown to be associated with the muscle outcome variables in this cohort^38,45^ or others^46,47^ and which were also hypothesized to be associated with statin use.

We included knee OA given its importance to proximal lower limb performance and intrusive body pain and because it is also associated with statin use^48^. We used the American College of Rheumatology criteria for the clinical diagnosis of idiopathic knee OA age 53, namely knee pain in either knee on most days for at least 1 month in the last year and at least two of the following: stiffness, crepitus, bony tenderness and bony enlargement. These items were assessed through a clinical examination conducted by a trained research nurse; further details have been published elsewhere^49^.

Information on life time cigarette smoking up to age 60–64 was classified into three categories: never, former and current smoker. Height and weight were measured by the research nurse according to standard protocols at a home visit at age 53, and body mass index (BMI) was calculated (kg/m^2^). Own occupational class was chosen to represent adult SEC based on the study member’s occupation at age 53 (or at 43, 36 or 26 years if missing at 53 years, n = 54). The Registrar General’s six-group social classification was collapsed into three groups: professional and intermediate (I and II); skilled non-manual (IIINM) or skilled manual (IIIM); and semi-skilled and unskilled manual (IV and V). The highest educational qualification obtained by age 26 was collapsed into three groups: degree level or advanced secondary qualifications (or equivalents); ordinary secondary level (or equivalents); no formal qualifications.

### Statistical Analysis

All statistical analyses were conducted using R (R Foundation for Statistical Computing, Vienna, Austria. http://www.R-project.org/).

There were 1556 study members with complete data on all outcomes and covariates. The characteristics of these men and women were compared using chi-squared tests and analysis of variance (ANOVA) as appropriate. The associations of statin use and 25-hydroxyvitamin D status with each muscle outcome were investigated in the combined sample of men and women as there was no evidence for interactions between sex and statin use with any of the outcomes. We used logistic regression for binary outcomes (intrusive body pain, difficulty going up and down stairs and difficulty gripping) and linear regression for continuous outcomes (chair rise speed and grip strength). In the first set of models, we examined the associations of statin use and 25-hydroxyvitamin D with each of the muscle outcomes separately, adjusted only for sex. In the second set of models, we tested for interactions between statin use and 25-hydroxyvitamin D status using likelihood ratio tests with 25-hydroxyvitamin D first modelled categorically and then rerun modelled continuously. This was to formally examine whether low 25-hydroxyvitamin D exacerbated the risk of muscle related outcomes as hypothesized a priori. We then further adjusted the models in which 25-hydroxyvitamin D was modelled categorically for inflammatory factors, BMI and knee OA, and finally for smoking status and adult SEC.

In sensitivity analyses we compared the estimates from the sex adjusted models in the sample with complete data and in the samples (maximum n = 2380) for whom statin use and at least one muscle related outcome was known.

### Transparency declaration

NS affirms that the manuscript is an honest, accurate, and transparent account of the study being reported and that no important aspects of the study have been omitted.

### Data sharing statement

Bona fide researchers can apply to access the NSHD data via a standard application procedure (further details available at: http://www.nshd.mrc.ac.uk/data.aspx doi:﻿10.5522/NSHD/Q101; doi:10.5522/NSHD/Q102).

## Results

A fifth of participants (19.9%) were taking statins (Table 1) with men more likely to be taking statins than women (Men 24.5%, Women 15.7%, Chi-squared test of sex difference p < 0.001). Compared with men, women reported more intrusive body pain and, difficulties going up and down stairs and gripping heavy objects in addition to slower chair rise speed and weaker grip strength.

**Table 1:** Characteristics of the sample of 1556 men and women in the MRC National Survey of Health and Development at age 60–64 with complete data.

	Men	Women	Men		Women	
			No Statin	Statin	No Statin	Statin
Total n in each group %	734	822	554	180	693	129
	47.2	52.8	75.5	24.5	84.3	15.7
**Outcomes**						
Intrusive body pain	32.6	43.6	30	40.6	41.3	55.8
Difficulty going up/down stairs	12.3	20.8	8.7	23.3	18.5	33.3
Difficulty gripping heavy objects	7.4	28.5	6.5	10	28.1	30.2
Chair Rise Speed (stands/min)	26.1	25.3	26.5	25	25.6	23.6
SD	(7.1)	(8.3)	(7.1)	(7.1)	(8.5)	(6.9)
Grip Strength max of 6 (kg)	45.3	24.7	45.4	44.9	24.8	24.3
SD	(11.9)	(7.9)	(12.2)	(11.1)	(8)	(7.9)
**Inflammatory Markers & 25-hydroxyvitamin D**						
IL6 (log pg/ml)	0.7	0.7	0.7	0.9	0.6	0.9
SD	(0.7)	(0.7)	(0.7)	(0.7)	(0.7)	(0.6)
CRP (log mg/l)	0.8	0.8	0.7	0.8	0.8	0.9
SD	(0.8)	(0.9)	(0.8)	(0.9)	(0.9)	(0.8)
25-hydroxyvitamin D						
Normal >20 ng/l	30.4	33.5	32.3	24.4	34.3	28.7
Insufficient 13–20 ng/l	34.2	33.6	32.7	38.9	32	41.9
Deficient <13 ng/l	35.4	33	35	36.7	33.6	29.5
**Covariates**						
BMI age 53 (kg/m^2^)	27.2	26.9	26.7	28.7	26.5	29.4
SD	(3.7)	(5)	(3.5)	(4)	(4.6)	(6.2)
Knee OA at 53 (Yes)	6.1	11.6	5.2	8.9	10.2	18.6
Education (by 26 years)						
None or Sub-GCE	35.4	38.1	32.1	45.6	36.2	48.1
O-level	14.7	28.1	14.8	14.4	28	28.7
A level/Degree or higher	49.9	33.8	53.1	40	35.8	23.3
SEC (age 53)						
I or II	57.1	40.1	58.1	53.9	41	35.7
IIINM or IIIM	32.8	43.7	31.6	36.7	44.2	41.1
IV or V	10.1	16.2	10.3	9.4	14.9	23.3
Cigarette Smoking						
Current	10.5	11.6	11	8.9	10.4	17.8
Exsmoker	60.9	53.8	58.7	67.8	54.4	50.4
Never	28.6	34.7	30.3	23.3	35.2	31.8

Compared with those not taking a statin, statin users reported more intrusive body pain and reduced proximal limb function (as indicated by slower chair rise speed and increased prevalence of reported difficultly going up/downstairs). Statin users also had higher mean levels of IL-6, higher mean BMI and were more likely to have knee OA (women only).

### Intrusive Body Pain

**Figure 1:**
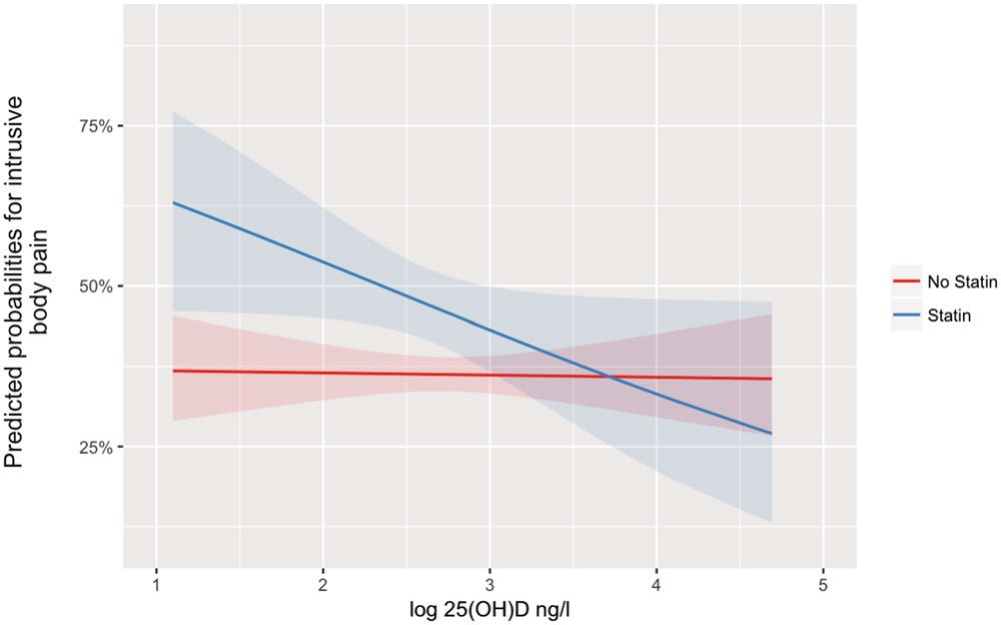
Interaction between statin use and log 25-hydroxyvitamin D (modelled continuously) in association with intrusive body pain (p-value for interaction = 0.09).

Statin use, but not 25-hydroxyvitamin D, was associated with greater odds of reporting intrusive body pain (Table 2, Model A). There was evidence of an interaction (p = 0.003) between statin use and 25-hydroxyvitamin D such that statin use was associated with greater odds of reporting intrusive body pain in the insufficient and deficient 25-hydroxyvitamin D groups but not in the normal group when 25-hydroxyvitamin D was modelled categorically (Model B). This interaction was also evident when 25-hydroxyvitamin D was modelled continuously (see Fig. 1). The estimates for statin use in the insufficient and deficient 25-hydroxyvitamin D groups were increasingly attenuated after adjusting first for inflammation (Model C), then BMI and knee OA (Model D) and finally smoking and SEC (Model E); Statin use in the insufficient group remained associated with intrusive body pain in the fully adjusted model (OR = 1.9, 95% CI 1.2 to 3.1).

**Table 2:** Odds ratios of intrusive body pain by statin use and 25-hydroxyvitamin D status, adjusted for inflammatory markers, BMI, knee OA, education, SEC and smoking (n = 1556).

	Model A (Sex adjusted only)			Model B (Mutually adjusted with interaction)			Intrusive Body Pain			Model D = Model C + BMI & Knee OA			Model E = Model D + SEC, education & smoking^†^		
							Model C = Model B + inflammatory markers								
	OR	95% CI	p value	OR	95% CI	p value	OR	95% CI	p value	OR	95% CI	p value	OR	95% CI	p value
Statin Use	1.7	(1.3,2.2)	<0.001												
**25-hydroxyvitamin D**															
Overall effect			0.598												
Normal >20 ng/l	1.0														
Insufficient 13–20 ng/l	1.0	(0.8,1.3)													
Deficient <13 ng/l	1.1	(0.9,1.4)													
Statin Use (vs non use) by 25-hydroxyvitamin D (Interaction*)															
Statin Use in those with Normal >20 ng/l				0.8	(0.5,1.4)	0.481	0.8	(0.5,1.3)	0.383	0.7	(0.4,1.3)	0.276	0.7	(0.4,1.2)	0.246
Statin Use in Insufficient 13–20 ng/l				2.6	(1.7,4.1)	<0.001	2.5	(1.6,3.9)	<0.001	2.0	(1.3,3.2)	0.002	1.9	(1.2,3.1)	0.005
Statin Use in Deficient<13 ng/l				1.8	(1.2,2.8)	0.004	1.8	(1.2,2.7)	0.006	1.5	(1.0,2.3)	0.059	1.4	(0.9,2.2)	0.106
IL6 (log pg/ml)							1.3	(1.1,1.5)	0.004	1.3	(1.1,1.5)	0.010	1.2	(1.0,1.5)	0.033
CRP (log mg/l)							1.3	(1.1,1.5)	<0.001	1.2	(1.0,1.4)	0.028	1.2	(1.0,1.3)	0.060
BMI at 53 (per 1 kg/m2 increase)										1.1	(1.0,1.1)	<0.001	1.1	(1.0,1.1)	<0.001
Knee OA at 53 (Yes vs No)										2.7	(1.9,4.0)	<0.001	2.7	(1.8,4.0)	<0.001
Sex (Men vs Women)				1.7	(1.4,2.1)	<0.001	1.7	(1.4,2.1)	<0.001	1.6	(1.3,2.0)	<0.001	1.6	(1.3,2.0)	<0.001

^†^SEC, education & smoking not shown.

^•^p-value = 0.003 for the interaction between statin use and 25-hydroxyvitamin D in Model B (formally tested using a likelihood ratio test comparing models with and without an interaction between 25-hydroxyvitamin D and statin use).

### Limb function

#### Difficulty going up/down stairs

**Figure 2:**
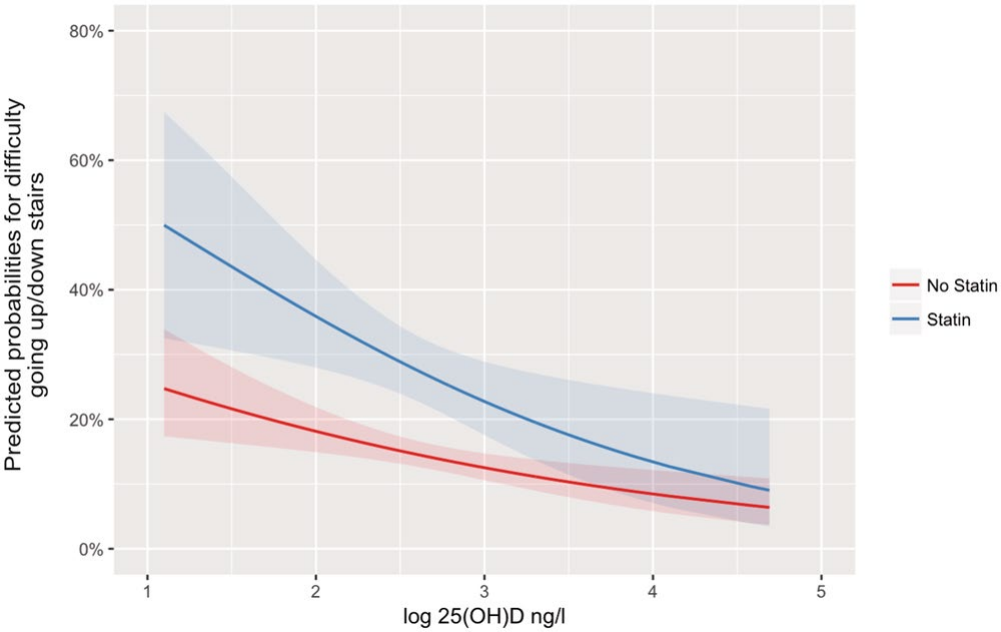
Interaction effect of statin use and log 25-hydroxyvitamin D (modelled continuously) in association with difficulty going up/down stairs (p-value for interaction = 0.48).

Statin use and being in the deficient 25-hydroxyvitamin D group were both associated with greater odds of difficulty going up/down stairs (Table 3 Model A). There was some evidence of an interaction between statin use and 25-hydroxyvitamin D (p = 0.078) such that statin use was associated with greater odds of difficulty with stairs in the insufficient and deficient but not the normal 25-hydroxyvitamin D groups when it was modelled categorically (Model B). There was no evidence of a statistically significant interaction when 25-hydroxyvitamin D was modelled continuously (see Fig. 2). These estimates were increasingly attenuated after adjustment for each set of covariates, although statin use remained associated with greater odds of difficulty with stairs in the fully adjusted model (Model E), both in the insufficient group (OR = 2.1, 95% CI 1.2 to 3.8, p = 0.012), and in the deficient group (OR = 2.1, 95% CI 1.2 to 3.5, p = 0.0051).

**Table 3:** Odds ratios of difficulty going up/down stairs by statin use and 25-hydroxyvitamin D status, adjusted for inflammatory markers, BMI, knee OA, education, SEC and smoking (n = 1556).

	Model A (Sex adjusted only)			Model B (Mutually adjusted with interaction)			Difficulty going up stairs			Model D = Model C + BMI & Knee OA			Model E = Model D + SEC, education & smoking^†^		
							Model C = Model B + inflammatory markers								
	OR	95% CI	p value	OR	95% CI	p value	OR	95% CI	p value	OR	95% CI	p value	OR	95% CI	p value
Statin Use	2.6	(1.9,3.5)	<0.001												
**25-hydroxyvitamin D**															
Overall effect			0.003												
Normal >20 ng/l	1.0														
Insufficient 13–20 ng/l	1.0	(0.7,1.5)													
Deficient <13 ng/l	1.6	(1.2,2.3)													
**Statin Use (vs non use) by 25-hydroxyvitamin D (Interaction)**															
Statin Use in those with Normal >20 ng/l				1.4	(0.7,2.6)	0.292	1.3	(0.7,2.5)	0.378	1.2	(0.6,2.3)	0.586	1.1	(0.5,2.2)	0.749
Statin Use in Insufficient 13–20 ng/l				3.6	(2.1,6.1)	<0.001	3.4	(1.9,5.9)	<0.001	2.4	(1.3,4.2)	0.003	2.1	(1.2,3.8)	0.012
Statin Use in Deficient <13 ng/l				2.7	(1.7,4.3)	<0.001	2.8	(1.7,4.5)	<0.001	2.2	(1.3,3.6)	0.003	2.1	(1.2,3.5)	0.005
IL6 (log pg/ml)							1.4	(1.1,1.7)	0.007	1.3	(1.0,1.7)	0.022	1.3	(1.0,1.6)	0.049
CRP (log mg/l)							1.6	(1.3,1.9)	<0.001	1.4	(1.1,1.7)	0.001	1.3	(1.1,1.6)	0.006
BMI at 53 (per 1 kg/m^2^ increase)										1.1	(1.1,1.1)	<0.001	1.1	(1.1,1.1)	<0.001
Knee OA at 53 (Yes vs No)										3.6	(2.4,5.3)	<0.001	3.5	(2.3,5.3)	<0.001
Sex (Men vs Women)				2.1	(1.6,2.9)	<0.001	2.2	(1.6,2.9)	<0.001	1.9	(1.4,2.6)	<0.001	1.8	(1.3,2.5)	<0.001

^†^SEC, education & smoking not shown.

*p-value = 0.078 for the interaction between statin use and 25-hydroxyvitamin D in Model B (formally tested using a likelihood ratio test comparing models with and without an interaction between 25-hydroxyvitamin D and statin use).

#### Difficulty gripping heavy objects

Neither statin use or 25-hydroxyvitamin D were associated with the odds of reporting difficulty gripping heavy objects in the sex-adjusted model, or in any subsequent regression model (Supplementary Table 1). There was weak evidence of an interaction between statin use and 25-hydroxyvitamin D (p = 0.093) such that in Model B statin use was associated with greater odds of difficulty gripping heavy objects in the insufficient group but not the deficient or the normal 25-hydroxyvitamin D groups. There was no evidence of a statistically significant interaction when 25-hydroxyvitamin D was modelled continuously (see Supplementary Figure 1).

#### Chair Rise speed

Statin use and being in the lower 25-hydroxyvitamin D groups were associated with slower chair rise speed (see Table 4, Model A). The differences in chair rise speed were greatest in the insufficient and deficient 25-hydroxyvitamin D groups but there was no formal statistical evidence of an interaction when 25-hydroxyvitamin D was modelled categorically (p = 0.32) or continuously. The associations were fully attenuated after adjustment for all the covariates; adjusting for BMI and knee OA reduced the estimates the most.

**Table 4:** Difference in mean chair rise speed (stands/minute) by statin use and 25-hydroxyvitamin D status, adjusted for inflammatory markers, BMI, knee OA, education, SEC and smoking (n = 1556).

	Model A (Sex adjusted only)			Model B (Mutually adjusted with interaction)			Chair Rise Speed/min			Model D = Model C + BMI & Knee OA			Model E = Model D + SEC, education & smoking^†^		
							Model C = Model B + inflammatory markers								
	Regression coefficient	95% CI	p value	Regression coefficient	95% CI	p value	Regression coefficient	95% CI	p value	Regression coefficient	95% CI	p value	Regression coefficient	95% CI	p value
Statin Use	−1.7	(−2.7, −0.7)	<0.001												
**25-hydroxyvitamin D**															
Overall effect			0.025												
Normal > 20 ng/l	0														
Insufficient 13–20 ng/l	−0.6	(−1.6, 0.3)													
Deficient <13 ng/l	−1.3	(−2.3, −0.4)													
**Statin Use (vs non use) by 25-hydroxyvitamin D (Interaction)**															
Statin Use in those with Normal > 20 ng/l				−0.5	(−2.3, 1.4)	0.605	−0.4	(−2.2, 1.5)	0.702	0.0	(−1.8, 1.8)	0.968	0.1	(−1.7, 1.8)	0.952
Statin Use in Insufficient 13–20 ng/l				−2.3	(−4.0, −0.7)	0.006	−2.0	(−3.7, −0.4)	0.017	−1.3	(−2.9, 0.4)	0.128	−1.0	(−2.6, 0.7)	0.245
Statin Use in Deficient <13 ng/l				−1.8	(−3.4, −0.2)	0.023	−1.7	(−3.2, −0.1)	0.033	−1.0	(−2.5, 0.5)	0.207	−0.8	(−2.3, 0.7)	0.302
IL6 (log pg/ml)							−0.9	(−1.6, −0.3)	0.004	−0.8	(−1.4, −0.2)	0.014	−0.7	(−1.3, −0.1)	0.027
CRP (log mg/l)							−1.0	(−1.5, −0.5)	<0.001	−0.7	(−1.2, −0.1)	0.013	−0.5	(−1.0, 0.0)	0.058
BMI at 53 (per 1 kg/m2 increase)										−0.2	(−0.3, −0.1)	<0.001	−0.2	(−0.3, −0.1)	<0.001
Knee OA at 53 (Yes vs No)										−2.4	(−3.8, −1.1)	<0.001	−2.2	(−3.6, −0.9)	0.001
Sex (Men vs Women)				−1.0	(−1.8, −0.2)	0.010	−1.0	(−1.7, −0.2)	0.012	−0.9	(−1.6, −0.1)	0.026	−0.5	(−1.3, 0.2)	0.179

^†^SEC, education & smoking not shown.

*p-value = 0.32 for the interaction between statin use and 25-hydroxyvitamin D in Model B (formally tested using a likelihood ratio test comparing models with and without an interaction between 25-hydroxyvitamin D and statin use).

#### Grip strength

There was no association between statin use and grip strength in the sex adjusted model (difference in mean grip strength (kg) = −0.5, 95% CI −1.8 to 0.8, p = 0.4), or in any subsequent regression models (Supplementary Table 2). There was no evidence of an interaction between statin use and 25-hydroxyvitamin D on grip strength p = 0.59).

#### Sensitivity Analysis

There was no difference in prevalence estimates of statin use when comparing the sub-sample with complete data (n = 1556) (19.9%) with the larger sample for whom statin use and at least one muscle related outcome was known (n = 2380) (20.7%). While there were some differences between the analytical sample and the sample of study members who were assessed at age 60–64 but did not have complete data and so were excluded from our analyses (the analytical sample had better physical performance, slightly lower mean BMI and were more likely to have higher educational levels), the estimates from sex adjusted models rerun using the maximum available samples were similar to the estimates reported.

## Discussion

In a nationally representative British population, statin use was associated in sex-adjusted models with increased odds of intrusive body pain and difficulty climbing stairs and with slower chair rise speed. The associations were strongest in those with insufficient or deficient 25-hydroxyvitamin D status. For chair rise speed, these associations were fully accounted for by inflammation, prior BMI, knee OA, smoking and SEC. For intrusive body pain and difficulties with stairs, there remained associations with statin use for those of insufficient and/or deficient 25-hydroxyvitamin D status in fully-adjusted models.

Previous studies^9–13,15–18^ have typically focused on one or another of the broad range of adverse side effects of statins. We instead examined a range of chronic muscle related outcomes including proximal and distal limb function (both self-reported and performance-based), and intrusive pain. Importantly, consistent with others^18^, we find no association between statin use and grip strength. Muscle related disorders tend to affect the proximal rather than distal muscles^50^ adding biological plausibility to our results. Our results support the view that the relationship between 25-hydroxyvitamin D status and health outcomes is non linear and is subject to a threshold effect^43^. This is evidenced by the weaker or absence of an interaction between 25-hydroxyvitamin D status and statin use when 25-hydroxyvitamin D is modeled continuously rather than categorically. This is consistent with clinical recommendations which typically use a threshold for treatment.

For intrusive body pain, the associations with statin use were only apparent in those with insufficient 25-hydroxyvitamin D status. These associations remained, albeit weakened, after adjustment for covariates. Previous cross sectional studies have suggested the involvement of 25-hydroxyvitamin D in statin related myalgia. Data from the NHANES, report greater myalgia in statin users in the low 25-hydroxyvitamin D groups^23^. However in a more select group of statin users - those who wished to switch to another statin - there did not appear to be an association between 25-hydroxyvitamin D status and myalgia^51^. In a small series of 128 patients^25^ with statin induced myalgia, 25-hydroxyvitamin D was significantly lower in statin users than non users. Importantly, in this study, the statin induced myalgia responded to 25-hydroxyvitamin D replacement in 92% of the cases. It should be noted that low 25-hydroxyvitamin D status is itself a cause of myopathy and myalgia^50^.

There was weak evidence in this study, that statin use was associated with difficulty going up and down stairs only for those with insufficient and/or deficient 25-hydroxyvitamin D status. Again these associations were only partially explained by the covariates. Statin use was associated with poorer objective motor performance (as indicated by slower chair rise speed) but the differences did not vary significantly across the 25-hydroxyvitamin D groups; and these associations were fully attenuated after adjustment for BMI, knee OA, smoking and SEC. There is conflicting evidence on whether statin use is associated with poor motor performance. Gray *et al*. found no differences between statin users and non users in a battery of tests assessed repeatedly over six years including 6-min walk test performance, repeated chair stands and grip strength (before and after adjustment for age, ethnicity, education, BMI, alcohol consumption, systolic and diastolic blood pressure, self-reported health, number of antihypertensive medications, diabetes mellitus, depressive symptoms, history of CHD, and hormone trial participation)^52^. Statin use did not substantially increase decline in gait speed in community-dwelling older adults (before and after adjustment for demographic characteristics, health-related behaviors, health status, and access to health care^53^). Some studies, such as the Three City Cohort (French cities; Bordeaux, Dijon, and Montpellier), even find that statin users have better physical performance than non users^54^. Notably however, 25-hydroxyvitamin D (or inflammatory markers and previous knee OA and SEC) are not typically included in these studies. Small interventional studies suggest that 25-hydroxyvitamin D supplementation can ameliorate statin induced intolerance of myopathy (and myalgia)^55,56^.

Given our findings, both the inverse associations between 25-hydroxyvitamin D status and certain muscle-related outcomes, and evidence that 25-hydroxyvitamin D status may modify the associations of statin use on these outcomes needs to be further investigated. Ideally a trial is required to address whether treatment of a low 25-hydroxyvitamin D prior to starting a statin prevents myalgia and any adverse change in proximal motor performance. Such studies may be redundant if a broader strategy to treat low 25-hydroxyvitamin D status in the general population is adopted. Pragmatically some would suggest that a low 25-hydroxyvitamin D is sufficient grounds for replacement given the pleiotropic effects 25-hydroxyvitamin D may have on other outcomes including cognitive function, cardiac disease and bone^41,57^.

What are the implications for clinical practice? As stated above, a trial may be required but may be redundant if clinicians feel there is sufficient grounds to treat low 25-hydroxyvitamin D due to the broad health benefits^43^. While it is recognised that low 25-hydroxyvitamin D is a risk factor for statin related adverse side effects^58,59^, we are not aware of a consensus statement regarding best practice with regards to statin use. The first author’s personal practice (NS) is to check 25-hydroxyvitamin D when considering a statin – the timing of replacement and statin initiation depends on the clinical situation; and to check and replace 25-hydroxyvitamin D in patients who appear to be intolerant of statins or report chronic side effects. No doubt practice varies depending upon the setting (primary versus secondary care) and the patient population. Clinicians should be aware of the possible relationship between low 25-hydroxyvitamin D and adverse side effects of statin use, and investigate and treat based upon local practice and individual patient circumstances.

In RCTs, what constitutes a statin related adverse effect is poorly defined and varies between trials. In 26 trials, broadly defined ‘muscle problems’ occurred in 12.7% of statin users versus 12.4% in the placebo group^60^. Trial data focus almost exclusively on the more acute side effects of statins (i.e., acute muscle inflammation): out of 42 trials, 16 do not even report the frequency of the more insidious chronic muscle problems. Only one trial asked about chronic muscle problems^61^. Trials may not be structured to identify these adverse effects. 25-hydroxyvitamin D status is not typically considered in trials but one would expect low 25-hydroxyvitamin D status to be balanced across treatment and placebo groups. Nevertheless, it may be prudent for future statin trials to assess 25-hydroxyvitamin D status and incorporate it into the randomization process.

Another relevant aspect of RCTs is that patients with severe hypertension, chronic obstructive pulmonary disease (COPD), renal disease and poorly controlled diabetes are typically excluded; and these patients may be more likely to have low 25-hydroxyvitamin D status^62–64^. A strength of NSHD is that the sample includes individuals with a range of health conditions and remains broadly representative of the population born in mainland Britain straight after the second world war^65^. However, a third of participants had one or more missing variables which may have affected its representativeness, although the proportion of statin users and the associations we observed between statin and non statin users were similar in the maximum and the complete data samples. Further, statin use and 25-hydroxyvitamin D status was comparable to other representative samples of those in early old age^66,67^. Nevertheless, our findings need to be replicated in older and younger cohorts to see whether they are generalizable to the whole adult population.

Another strength of this study is that our sample was homogeneous for age which enabled us to examine associations free from the confounding effect of age. In samples heterogeneous for age, it is consistently found to be one of the strongest predictors of statin related adverse effects^10,11^. Given low 25-hydroxyvitamin D status increases with age^68^ and is associated with telomere length^69^, further work is needed to explore whether this partly explains the age related association between statin use and muscle-related outcomes.

This study also has additional limitations. First, statin use was self-reported and we do not have information on the type of statin, the dose or its duration. Second, we cannot directly assess pre and post statin function and cannot comment on acute adverse side effects such as myositis. Third, we do not have information on how many study members have tried and stopped statins in the past. Fourth, residual confounding may remain from unmeasured factors, especially as the covariates used did at least partly attenuate the associations observed between statin use and the muscle related outcomes.

In conclusion, we find that statin use was associated with a range of non acute muscle related outcomes in men and women in early old age. These associations were confined or more apparent in those with low 25-hydroxyvitamin D status, and partly explained by other risk factors such as BMI and knee OA. Replication in older and younger populations is required given recent NICE guidelines that call for more widespread use of statins. Although a trial is required to determine if treatment of low 25-hydroxyvitamin D prevents statin related adverse effects, if there are clear clinical grounds for treating a low 25-hydroxyvitamin D then it may be reasonable to do so before starting statin treatment. The recommendations from the UK Government 25-hydroxyvitamin D group are awaited.

## Electronic supplementary material

Supplementary Information

## References

[1] Efficacy and safety of LDL-lowering therapy among men and women: meta-analysis of individual data from 174, 000 participants in 27 randomised trials. The Lancet **385**, 1397–1405 (2015). 10.1016/S0140-6736(14)61368-4 25579834

[2] Baigent C (2010). Efficacy and safety of more intensive lowering of LDL cholesterol: a meta-analysis of data from 170,000 participants in 26 randomised trials. Lancet.

[3] Cardiovascular disease: risk assessment and reduction, including lipid modifification. nice.org.ukguidancecg 1–50 (2016).

[4] McPherson K (2014). Concerns about the latest NICE draft guidance on statins. BMJ.

[5] Ben G, Smeeth L (2014). Mass treatment with statins. BMJ.

[6] Majeed A, Molokhia M (2014). Urgent need to establish the true incidence of the side effects of statins. BMJ.

[7] Finegold JA, Manisty CH, Goldacre B, Barron AJ, Francis DP (2014). What proportion of symptomatic side effects in patients taking statins are genuinely caused by the drug? Systematic review of randomized placebo-controlled trials to aid individual patient choice. Eur J Prev Cardiol.

[8] Zhang H (2013). Discontinuation of statins in routine care settings: a cohort study. Ann Intern Med.

[9] Cohen JD, Brinton EA, Ito MK, Jacobson TA (2012). Understanding Statin Use in America and Gaps in Patient Education (USAGE): an internet-based survey of 10,138 current and former statin users. J Clin Lipidol.

[10] Buettner C, Davis RB, Leveille SG, Mittleman MA, Mukamal KJ (2008). Prevalence of musculoskeletal pain and statin use. J Gen Intern Med.

[11] Nichols GA, Koro CE (2007). Does statin therapy initiation increase the risk for myopathy? An observational study of 32,225 diabetic and nondiabetic patients. Clin Ther.

[12] Bruckert E, Hayem G, Dejager S, Yau C, Bégaud B (2005). Mild to moderate muscular symptoms with high-dosage statin therapy in hyperlipidemic patients–the PRIMO study. Cardiovasc Drugs Ther.

[13] Hippisley-Cox J, Coupland C (2010). Individualising the risks of statins in men and women in England and Wales: population-based cohort study. Heart.

[14] Smeeth L, Douglas I, Hall AJ, Hubbard R, Evans S (2009). Effect of statins on a wide range of health outcomes: a cohort study validated by comparison with randomized trials. Br J Clin Pharmacol.

[15] Agostini JV (2007). Effects of statin use on muscle strength, cognition, and depressive symptoms in older adults. J Am Geriatr Soc.

[16] Giri J (2006). Statin use and functional decline in patients with and without peripheral arterial disease. J. Am. Coll. Cardiol..

[17] Swiger KJ, Manalac RJ, Blaha MJ, Blumenthal RS, Martin SS (2014). Statins, mood, sleep, and physical function: a systematic review. Eur. J. Clin. Pharmacol..

[18] Witham MD (2014). ACE inhibitors, statins and thiazides: no association with change in grip strength among community dwelling older men and women from the Hertfordshire Cohort Study. Age Ageing.

[19] Henderson, R. M. *et al*. Effect of Statin Use on Mobility Disability and its Prevention in At-risk Older Adults: The LIFE Study. *J. Gerontol. A Biol. Sci. Med. Sci*, doi:10.1093/gerona/glw057 (2016). 10.1093/gerona/glw057 PMC5055646 26988662

[20] Hill TR (2014). Vitamin D status is poor in the UK. BMJ.

[21] Hyppönen E, Power C (2007). Hypovitaminosis D in British adults at age 45 y: nationwide cohort study of dietary and lifestyle predictors. Am. J. Clin. Nutr..

[22] Palamaner Subash Shantha G, Ramos J, Thomas-Hemak L, Pancholy SB (2014). Association of vitamin D and incident statin induced myalgia–a retrospective cohort study. PLoS ONE.

[23] Morioka TY, Lee AJ, Bertisch S, Buettner C (2015). Vitamin D status modifies the association between statin use and musculoskeletal pain: a population based study. Atherosclerosis.

[24] Michalska-Kasiczak M (2015). Analysis of vitamin D levels in patients with and without statin-associated myalgia - a systematic review and meta-analysis of 7 studies with 2420 patients. Int J Cardiol.

[25] Ahmed W (2009). Low serum 25 (OH) vitamin D levels (<32 ng/mL) are associated with reversible myositis-myalgia in statin-treated patients. Transl Res.

[26] Skaaby, T. *et al*. Longitudinal associations between lifestyle and vitamin D: A general population study with repeated vitamin D measurements. Endocrine 1–9, doi:10.1007/s12020-015-0641-7 (2015). 10.1007/s12020-015-0641-7 26024976

[27] Karras, S. *et al*. Hypovitaminosis D in pregnancy in the Mediterranean region: a systematic review. *Eur J Clin Nutr*, doi:10.1038/ejcn.2016.12 (2016). 10.1038/ejcn.2016.12 26931671

[28] Cesari M (2004). Inflammatory markers and physical performance in older persons: the InCHIANTI study. The Journals of Gerontology Series A: Biological Sciences and Medical Sciences.

[29] Volaklis KA (2015). Association between muscular strength and inflammatory markers among elderly persons with cardiac disease: results from the KORA-Age study. Clin Res Cardiol.

[30] Taaffe DR, Harris TB, Ferrucci L, Rowe J, Seeman TE (2000). Cross-sectional and prospective relationships of interleukin-6 and C-reactive protein with physical performance in elderly persons: MacArthur studies of successful aging. The Journals of Gerontology Series A: Biological Sciences and Medical Sciences.

[31] van Dooren, F. E. P. *et al*. Associations of low grade inflammation and endothelial dysfunction with depression – The Maastricht Study. Brain, Behavior, and Immunity 1–7, doi:10.1016/j.bbi.2016.03.004 (2016). 10.1016/j.bbi.2016.03.004 26970354

[32] Hayden KE, Sandle LN, Berry JL (2015). Ethnicity and social deprivation contribute to vitamin D deficiency in an urban UK population. The Journal of Steroid Biochemistry and Molecular Biology.

[33] Kuh D (2011). Cohort profile: updating the cohort profile for the MRC National Survey of Health and Development: a new clinic-based data collection for ageing research. Int J Epidemiol.

[34] Brazier J, Roberts J, Deverill M (2002). The estimation of a preference-based measure of health from the SF-36. J Health Econ.

[35] Kuh D (2005). Grip Strength, Postural Control, and Functional Leg Power in a Representative Cohort of British Men and Women: Associations With Physical Activity, Health Status, and Socioeconomic Conditions. The Journals of Gerontology Series A: Biological Sciences and Medical Sciences.

[36] Martin, J., Meltzer, H. & Elliot, D. The prevalence of disability among adults. (HMSO Books, 1988).

[37] Hurst L (2013). Lifetime Socioeconomic Inequalities in Physical and Cognitive Aging. American Journal of Public Health.

[38] Mänty M, Kuh D, Cooper R (2015). Associations of Midlife to Late Life Fatigue With Physical Performance and Strength in Early Old Age. Psychosomatic Medicine.

[39] Kuh D (2006). Developmental origins of midlife grip strength: findings from a birth cohort study. The Journals of Gerontology Series A: Biological Sciences and Medical Sciences.

[40] Murray, E. T. *et al*. Overweight across the life course and adipokines, inflammatory and endothelial markers at age 60-64 years: Evidence from the 1946 birth cohort. *Int J Obes* (*Lond*), doi:10.1038/ijo.2015.19 (2015). 10.1038/ijo.2015.19 PMC4433551 25676237

[41] Institute of Medicine (US) Committee to Review Dietary Reference Intakes for Vitamin D and Calcium, Ross, A. C., Taylor, C. L., Yaktine, A. L. & Del Valle, H. B. Dietary Reference Intakes for Calcium and Vitamin D, doi:10.17226/13050 (2011). 21796828

[42] Braga, M., Simmons, Z., Norris, K. C., Ferrini, M. G. & Artaza, J. N. Vitamin D induces myogenic differentiation in skeletal muscle derived stem cells. Endocrine Connections EC–17–0008, doi:10.1530/EC-17-0008 (2017). 10.1530/EC-17-0008 PMC5424772 28174253

[43] Bischoff-Ferrari HA, Giovannucci E, Willett WC, Dietrich T, Dawson-Hughes B (2006). Estimation of optimal serum concentrations of 25-hydroxyvitamin D for multiple health outcomes. Am. J. Clin. Nutr..

[44] Sahota O (2014). Understanding vitamin D deficiency. Age and Ageing.

[45] Cooper R, Strand BH, Hardy R, Patel KV, Kuh D (2014). Physical capability in mid-life and survival over 13 years of follow-up: British birth cohort study. BMJ.

[46] Callisaya ML, Blizzard L, Schmidt MD, McGinley JL, Srikanth VK (2010). Ageing and gait variability–a population-based study of older people. Age and Ageing.

[47] Salonen MK (2011). Developmental Origins of Physical Fitness: The Helsinki Birth Cohort Study. PLoS ONE.

[48] Kadam UT, Blagojevic M, Belcher J (2013). Statin Use and Clinical Osteoarthritis in the General Population: A Longitudinal Study. J Gen Intern Med.

[49] Wills AK (2012). Life course body mass index and risk of knee osteoarthritis at the age of 53 years: evidence from the 1946 British birth cohort study. Ann Rheum Dis.

[50] Suresh E, Wimalaratna S (2013). Proximal myopathy: diagnostic approach and initial management. Postgrad Med J.

[51] Kurnik D (2012). Muscle pain and serum creatine kinase are not associated with low serum 25(OH) vitamin D levels in patients receiving statins. Clin Endocrinol (Oxf).

[52] Gray SL (2012). Statins, angiotensin-converting enzyme inhibitors, and physical performance in older women. J Am Geriatr Soc.

[53] Lo-Ciganic W-H (2015). Statin use and decline in gait speed in community-dwelling older adults. J Am Geriatr Soc.

[54] Dumurgier J, Singh-Manoux A, Tavernier B, Tzourio C, Elbaz A (2014). Lipid-lowering drugs associated with slower motor decline in the elderly adults. J. Gerontol. A Biol. Sci. Med. Sci..

[55] Khayznikov M (2015). Statin Intolerance Because of Myalgia, Myositis, Myopathy, or Myonecrosis Can in Most Cases be Safely Resolved by Vitamin D Supplementation. N Am J Med Sci.

[56] Glueck CJ (2011). Vitamin D deficiency, myositis-myalgia, and reversible statin intolerance. Curr Med Res Opin.

[57] Sanders KM, Scott D, Ebeling PR (2014). Vitamin D deficiency and its role in muscle-bone interactions in the elderly. Curr Osteoporos Rep.

[58] Patel J, Martin SS, Banach M (2016). Expert opinion: the therapeutic challenges faced by statin intolerance. Expert Opinion on Pharmacotherapy.

[59] Banach M (2015). Position paper Statin intolerance – an attempt at a unified definition. Position paper from an International Lipid Expert Panel. aoms.

[60] Ganga HV, Slim HB, Thompson PD (2014). A systematic review of statin-induced muscle problems in clinical trials. American Heart Journal.

[61] Kjekshus J (2007). Rosuvastatin in older patients with systolic heart failure. N Engl J Med.

[62] Mekov E (2015). Vitamin D Deficiency and Insufficiency in Hospitalized COPD Patients. PLoS ONE.

[63] Patel NM (2010). Vitamin D deficiency and anemia in early chronic kidney disease. Kidney International.

[64] Thomas GN (2012). Hyperglycaemia and vitamin D: a systematic overview. Curr Diabetes Rev.

[65] Stafford M (2013). Using a birth cohort to study ageing: representativeness and response rates in the National Survey of Health and Development. Eur J Ageing.

[66] Ford L, Graham V, Wall A, Berg J (2006). Vitamin D concentrations in an UK inner-city multicultural outpatient population. Ann. Clin. Biochem..

[67] Murphy C (2015). Statin use in adults at high risk of cardiovascular disease mortality: cross-sectional analysis of baseline data from The Irish Longitudinal Study on Ageing (TILDA). BMJ Open.

[68] Souberbielle, J.-C., Massart, C., Brailly-Tabard, S., Cavalier, E. & Chanson, P. Prevalence and determinants of vitamin D deficiency in healthy French adults: the VARIETE study. Endocrine 1–8, doi:10.1007/s12020-016-0960-3 (2016). 10.1007/s12020-016-0960-3 27106800

[69] Mazidi, M., Michos, E. D. & Banach, M. The association of telomere length and serum 25-hydroxyvitamin D levels in US adults: the National Health and Nutrition Examination Survey. aoms **1**, 61–65 (2017). 10.5114/aoms.2017.64714 PMC5206371 28144256

