# Publisher Correction: Associations of statin use with motor performance and myalgia may be modified by 25-hydroxyvitamin D: findings from a British birth cohort

**DOI:** 10.1038/s41598-018-20585-w

**Published:** 2018-02-20

**Authors:** Nikhil Sharma, Rachel Cooper, Diana Kuh, Imran Shah

**Affiliations:** MRC Unit for Lifelong Health and Ageing at UCL, 33 Bedford Place, London, WC1B 5JU UK

Correction to: *Scientific Reports* 10.1038/s41598-017-06019-z, published online 26 July 2017

Imran Shah was omitted from the author list in the original version of this Article. This has been corrected in the PDF and HTML versions of the Article.

