# Supplementary material for: Associations of statin use with motor performance and myalgia may be modified by 25-hydroxyvitamin D: findings from a British birth cohort
Source: Sci Rep. 2017 Jul 26;7:6578. doi: 10.1038/s41598-017-06019-z (PMC5529559; doi:10.1038/s41598-017-06019-z)
Supplement: Supplementary file 1 — Supplementary Information [file 41598_2017_6019_MOESM1_ESM.doc]

**Associations of statin use with motor performance and myalgia may be modified by 25-hydroxyvitamin D: findings from a British birth cohort.**

Nikhil Sharma Senior Clinical Researcher and Honorary Consultant Neurologist ^1^

Rachel Cooper Senior Lecturer and MRC Programme Leader Track ^1^

Imran Shah, Data Analyst ^1^

Diana Kuh Professor of Life Course Epidemiology and MRC Unit Director ^1^

^1^ MRC Unit for Lifelong Health and Ageing at UCL, 33 Bedford Place, London WC1B 5JU, UK

Supplementary Table 1: Odds ratio of difficulty gripping heavy objects by statin use and 25-hydroxyvitamin D status, adjusted for inflammatory markers, BMI, knee OA, education, SEC and smoking (n= 1556).

|  | |  | | |  |  | | | |  | **Difficulty gripping heavy objects** | | |  |  | | |  |  | | |  |
| --- | --- | --- | --- | --- | --- | --- | --- | --- | --- | --- | --- | --- | --- | --- | --- | --- | --- | --- | --- | --- | --- | --- |
|  | | **Model A (Sex adjusted only)** | | |  | **Model B (Mutually adjusted with interaction)** | | | |  | **Model C = Model B + inflammatory markers** | | |  | **Model D = Model C + BMI & Knee OA** | | |  | **Model E = Model D + SEC, education & smoking†** | | |  |
|  | | **OR** | **95% CI** | **p value** |  | **OR** | | **95% CI** | **p value** |  | **OR** | **95% CI** | **p value** |  | **OR** | **95% CI** | **p value** |  | **OR** | **95% CI** | **p value** |  |
|  | | | | | | | | | | | | | | | | | | | | | |  |
| Statin Use | | 1.2 | (0.9,1.7) | 0.211 |  |  | |  |  |  |  |  |  |  |  |  |  |  |  |  |  |  |
| **25-hydroxyvitamin D** | | | | | | | | | | | | | | | | | | | | | |  |
| Overall effect | |  |  | 0.149 |  |  |  | | | | | | | | | | | | | | | |
| Normal >20 ng/l | | 1.0 |  |  |  |  |  | | | | | | | | | | | | | | | |
| Insufficient 13-20ng/l | | 0.9 | (0.6,1.3) |  |  |  |  | | | | | | | | | | | | | | | |
| Deficient <13ng/l | | 1.2 | (0.9,1.7) |  |  |  |  | | | | | | | | | | | | | | | |
| **Statin Use (vs non use) by 25-hydroxyvitamin D (Interaction)** | | | | | | | | | | | | | | | | | | | | | |  |
| Statin Use in those with Normal >20 ng/l | |  |  |  |  | 0.7 | | (0.3,1.3) | 0.260 |  | 0.6 | (0.3,1.3) | 0.236 |  | 0.6 | (0.3,1.3) | 0.250 |  | 0.6 | (0.3,1.3) | 0.213 |  |
| Statin Use in Insufficient 13-20ng/l | |  |  |  |  | 1.8 | | (1.0,3.2) | 0.049 |  | 1.7 | (0.9,3.0) | 0.082 |  | 1.5 | (0.8,2.7) | 0.187 |  | 1.4 | (0.7,2.5) | 0.286 |  |
| Statin Use in Deficient <13ng/l | |  |  |  |  | 1.3 | | (0.8,2.2) | 0.312 |  | 1.3 | (0.7,2.1) | 0.364 |  | 1.2 | (0.7,2.0) | 0.563 |  | 1.1 | (0.6,1.8) | 0.809 |  |
|  |  | | | | | | | | | | | | | | | | | | | | | |
|  | | | | | | | | | | | | | | | | | | | | | |  |
| IL6 (log pg/ml) | |  |  |  |  |  | |  |  |  | 1.2 | (0.9,1.5) | 0.132 |  | 1.2 | (0.9,1.5) | 0.164 |  | 1.1 | (0.9,1.4) | 0.232 |  |
| CRP (log mg/l) | |  |  |  |  |  | |  |  |  | 1.2 | (1.0,1.5) | 0.019 |  | 1.2 | (1.0,1.4) | 0.065 |  | 1.1 | (1.0,1.4) | 0.140 |  |
| BMI at 53 (per 1 kg/m2 increase) | |  |  |  |  |  | |  |  |  |  |  |  |  | 1.0 | (1.0,1.0) | 0.465 |  | 1.0 | (1.0,1.0) | 0.551 |  |
| Knee OA at 53 (Yes vs No) | |  |  |  |  |  | |  |  |  |  |  |  |  | 1.9 | (1.3,2.9) | 0.002 |  | 1.9 | (1.2,2.8) | 0.003 |  |
| Sex (Men vs Women) | |  |  |  |  | 5.2 | | (3.8,7.2) | <0.001 |  | 5.2 | (3.8,7.2) | <0.001 |  | 5.0 | (3.6,7.0) | <0.001 |  | 5.0 | (3.6,7.0) | <0.001 |  |
| **† SEC, education & smoking not shown**  *** p-value=0.093 for the interaction between statin use and 25-hydroxyvitamin D in Model B (formally tested using a likelihood ratio test comparing models with and without an interaction between 25-hydroxyvitamin D and statin use)** | | | | | | | | | | | | | | | | | | | | | |  |

Supplementary Table 2: Difference in mean grip strength (kg) by statin use and 25-hydroxyvitamin D status, adjusted for inflammatory markers, BMI, knee OA, education, SEC and smoking (n= 1556).

|  | |  | | |  |  | | | |  | **Grip Strength** | | |  |  | | |  |  | | |  |
| --- | --- | --- | --- | --- | --- | --- | --- | --- | --- | --- | --- | --- | --- | --- | --- | --- | --- | --- | --- | --- | --- | --- |
|  | | **Model A (Sex adjusted only)** | | |  | **Model B (Mutually adjusted with interaction)** | | | |  | **Model C = Model B + inflammatory markers** | | |  | **Model D = Model C + BMI & Knee OA** | | |  | **Model E = Model D + SEC, education & smoking†** | | |  |
|  | | **Regression coefficient** | **95% CI** | **p value** |  | **Regression coefficient** | | **95% CI** | **p value** |  | **Regression coefficient** | **95% CI** | **p value** |  | **Regression coefficient** | **95% CI** | **p value** |  | **Regression coefficient** | **95% CI** | **p value** |  |
|  | | | | | | | | | | | | | | | | | | | | | |  |
| Statin Use | | -0.2 | ( -1.4, 1.1) | 0.764 |  |  | |  |  |  |  |  |  |  |  |  |  |  |  |  |  |  |
| **25-hydroxyvitamin D** | | | | | | | | | | | | | | | | | | | | | |  |
| Overall effect | |  |  | 0.426 |  |  |  | | | | | | | | | | | | | | | |
| Normal >20 ng/l | | 0 |  |  |  |  |  | | | | | | | | | | | | | | | |
| Insufficient Vit D 13-20ng/l | | 0.0 | ( -1.3, 1.2) |  |  |  |  | | | | | | | | | | | | | | | |
| Deficient <13ng/l | | -0.7 | ( -1.9, 0.5) |  |  |  |  | | | | | | | | | | | | | | | |
| **Statin Use (vs non use) by 25-hydroxyvitamin D (Interaction)** | | | | | | | | | | | | | | | | | | | | | |  |
| Statin Use in those with Normal >20 ng/l | |  |  |  |  | -0.9 | | ( -3.3, 1.5) | 0.449 |  | -0.8 | ( -3.1, 1.6) | 0.518 |  | -1.2 | ( -3.5, 1.2) | 0.328 |  | -1.1 | ( -3.4, 1.3) | 0.367 |  |
| Statin Use in Insufficient 13-20ng/l | |  |  |  |  | 0.7 | | ( -1.4, 2.8) | 0.512 |  | 1.0 | ( -1.1, 3.1) | 0.365 |  | 0.9 | ( -1.2, 3.1) | 0.398 |  | 1.2 | ( -1.0, 3.3) | 0.287 |  |
| Statin Use in Deficient <13ng/l | |  |  |  |  | -0.3 | | ( -2.4, 1.7) | 0.746 |  | -0.2 | ( -2.3, 1.8) | 0.819 |  | -0.4 | ( -2.5, 1.6) | 0.670 |  | -0.3 | ( -2.3, 1.7) | 0.784 |  |
|  |  | | | | | | | | | | | | | | | | | | | | | |
|  | | | | | | | | | | | | | | | | | | | | | |  |
| IL6 (log pg/ml) | |  |  |  |  |  | |  |  |  | -0.9 | ( -1.8, -0.1) | 0.023 |  | -1.0 | ( -1.8, -0.2) | 0.015 |  | -0.9 | ( -1.8, -0.1) | 0.023 |  |
| CRP (log mg/l) | |  |  |  |  |  | |  |  |  | -0.5 | ( -1.2, 0.1) | 0.124 |  | -0.6 | ( -1.3, 0.1) | 0.072 |  | -0.5 | ( -1.2, 0.2) | 0.160 |  |
| BMI at 53 (per 1 kg/m2 increase) | |  |  |  |  |  | |  |  |  |  |  |  |  | 0.1 | ( 0.0, 0.3) | 0.017 |  | 0.2 | ( 0.0, 0.3) | 0.010 |  |
| Knee OA at 53 (Yes vs No) | |  |  |  |  |  | |  |  |  |  |  |  |  | -2.3 | ( -4.0, -0.5) | 0.013 |  | -2.1 | ( -3.8, -0.3) | 0.023 |  |
| Sex (Men vs Women) | |  |  |  |  | -19.2 | | (-20.2,-18.2) | <0.001 |  | -19.2 | (-20.2,-18.2) | <0.001 |  | -19.1 | (-20.1,-18.1) | <0.001 |  | -18.8 | (-19.8,-17.7) | <0.001 |  |
| **† SEC, education & smoking not shown** | | | | | | | | | | | | | | | | | | | | | |  |

*** p-value=0.585 for the interaction between statin use and 25-hydroxyvitamin D in Model B (formally tested using a likelihood ratio test comparing models with and without an interaction between 25-hydroxyvitamin D and statin use)**


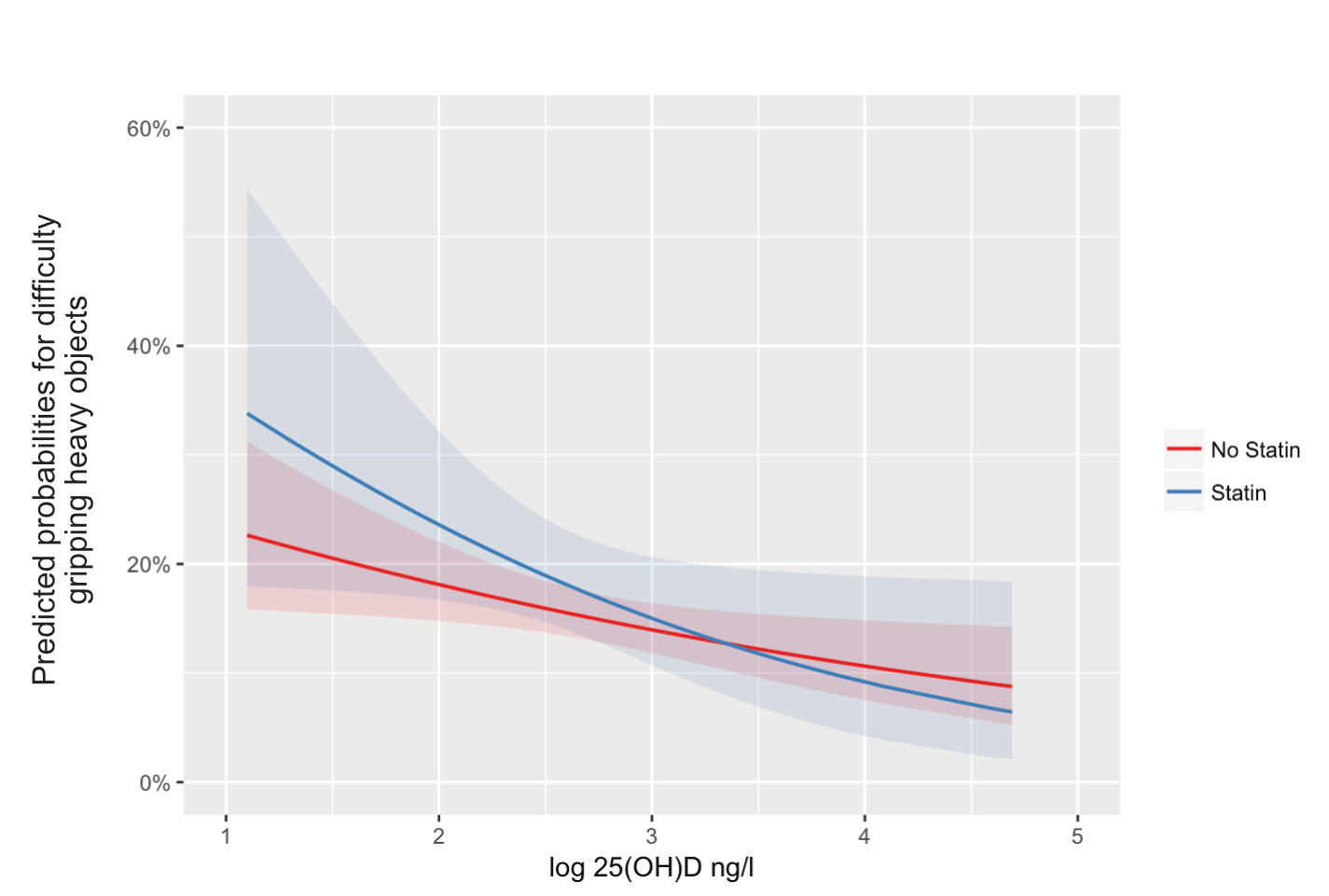


**Supplementary Figure 1: Interaction effect of statin use and log 25-hydroxyvitamin D (modelled continuously) in association with gripping heavy objects (p-value for interaction=0.41).**
